# Screen for Genetic Modifiers of *stbm* Reveals that Photoreceptor Fate and Rotation Can Be Genetically Uncoupled in the *Drosophila* Eye

**DOI:** 10.1371/journal.pone.0000453

**Published:** 2007-05-16

**Authors:** Tanya Wolff, Jake B. Guinto, Amy S. Rawls

**Affiliations:** Department of Genetics, Washington University School of Medicine, St. Louis, Missouri, United States of America; Ecole Normale Superieure, France

## Abstract

**Background:**

Polarity of the *Drosophila* compound eye arises primarily as a consequence of two events that are tightly linked in time and space: fate specification of two photoreceptor cells, R3 and R4, and the subsequent directional movement of the unit eyes of the compound eye, or ommatidia. While it is thought that these fates dictate the direction of ommatidial rotation, the phenotype of mutants in the genes that set up this polarity led to the hypothesis that these two events could be uncoupled.

**Methodology/Principal Findings:**

To definitively demonstrate these events are genetically separable, we conducted a dominant modifier screen to determine if genes, when misexpressed, could selectively enhance subclasses of mutant ommatidia in which the direction of rotation does not follow the R3/R4 cell fates, yet not affect the number of ommatidia in which rotation follows the R3/R4 cell fates. We identified a subset of P element lines that exhibit this selective enhancement. We also identified lines that behave in the opposite manner: They enhance the number of ommatidia that rotate in the right direction, but do not alter the number of ommatidia that rotate incorrectly with respect to the R3/R4 fates.

**Conclusions/Significance:**

These results indicate that fate and direction of rotation can be genetically separated, and that there are genes that act between R3/R4 fate specification and direction of ommatidial rotation. These data affirm what has been a long-standing assumption about the genetic control of ommatidial polarity.

## Introduction

The proximity of events in time and location during development poses an obstacle in establishing whether a later event is a direct consequence of a former, or if the events occur independently of one another. Independent events may be regulated sequentially, either by distinct sets of genes or by the same genes exhibiting their pleiotropic capacity to be recycled to regulate diverse events, further complicating analysis. Making these distinctions is a necessary prerequisite to studying a given developmental event in depth.

In the *Drosophila* compound eye, two events – the establishment of photoreceptor fates and a subsequent sophisticated morphogenetic movement of the photoreceptors – affect the same set of cells at essentially the same time in development. The tight coupling of these events in space and time has precluded the ability to establish whether the direction of the cellular movement is solely a consequence of the cell fates or if additional genes or pathways influence the movement. The screen described here reveals that these two events can be genetically uncoupled from one another.

**Figure 1:**
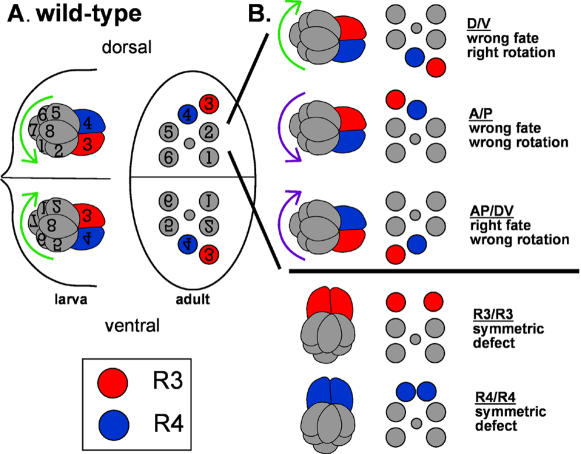
Derivation of wild-type and mutant ommatidial forms. (A) In wild-type imaginal discs, ommatidial precursors rotate 90° counterclockwise in the dorsal half of the eye and 90° clockwise in the ventral half (green arrows). Final adult forms, following rotation and additional morphological changes, are shown as trapezoids. (B) Corresponding mutant forms of ommatidial precursors and adult trapezoids from dorsal half of the eye are shown. D/V forms arise as a consequence of the wrong fate choice followed by the right direction of rotation with respect to those fates. A/P forms occur when the wrong fates are chosen but, unlike D/V inversions, ommatidial precursors subsequently rotate in the wrong direction with respect to the fates. AP/DV ommatidial result from the correct fate choice but wrong direction of rotation. Legend: photoreceptor R3 is denoted by red fill and R4 by blue fill. Green arrows indicate correct direction of rotation with respect to R3/R4 fates. Purple arrows indicate wrong direction of rotation. Anterior is to the right.

**Figure 2:**
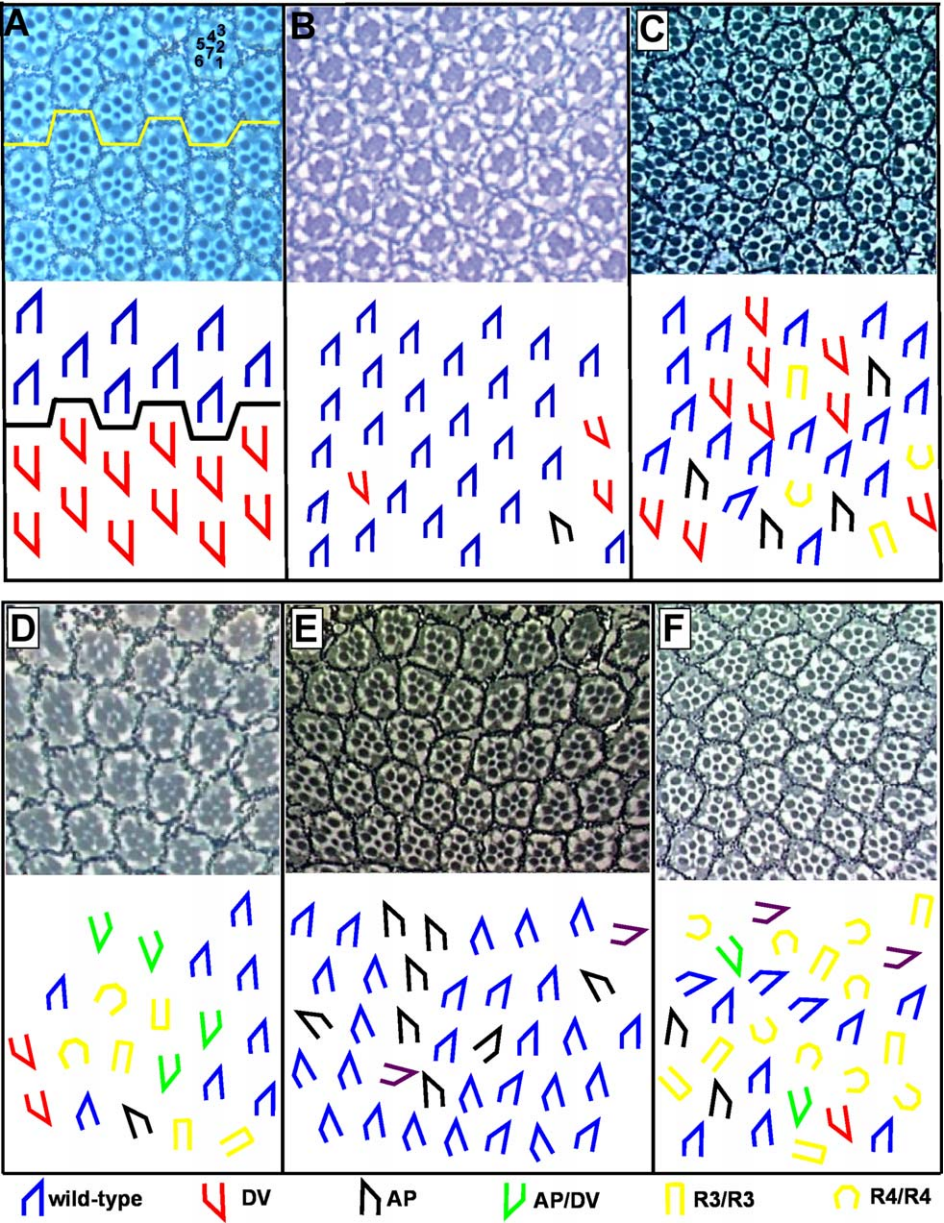
Modification of *sev-stbm* phenotype by GMREP lines. (A) Wild-type eyes have two ommatidial forms, dorsal (blue) and ventral (red); each class falls exclusively on the dorsal or ventral side of the equator (yellow/black line). (B) *sev-stbm* eyes have polarity defects in 11% of ommatidia. Three DV (red trapezoid) and one AP (black trapezoid) inversions are shown here. (C) A GMREP line that primarily enhances the number of DV defects in the *sev-stbm*/+ phenotype. (D) The number AP/DV (green trapezoid) defects is enhanced by the GMREP line shown here in a *sev-stbm*/+ background. (E) An eye representative of GMREP lines that enhance the AP-type defects in a *sev-stbm* background. (F) The modified eye shown in this panel has a marked increase in the number of R3/R3 and R4/R4 symmetric defects (yellow open rectangles and horseshoe shapes). Key: examples shown are for ommatidia in dorsal half of eye. Blue: wild-type; red: D/V inversion; black: A/P inversion; green: AP/DV inversion; yellow rectangle: R3/R3 symmetric ommatidia; yellow horseshoe: R4/R4 symmetric ommatidia.

The *Drosophila* compound eye consists of a precisely patterned, hexagonal array of roughly 800 unit eyes, or ommatidia (reviewed in [1]). Each ommatidium contains eight photoreceptors (R1–R8). The rhabdomeres, or photosensitive organelles of the photoreceptors, are arranged in characteristic trapezoids, with photoreceptor R3's rhabdomere occupying the “point” of the trapezoid (Figs. 1A adult, 2A). The *Drosophila* retina exhibits global polarity in that the trapezoids in the dorsal and ventral halves of the eye point in opposite directions (Figs. 1A adult, 2A). This polarized arrangement of the two chiral forms of ommatidia results in mirror symmetry across a dorsal/ventral (D/V) midline that runs horizontally across the eye and is known as the equator (Fig. 2A, yellow and black lines).

Polarity in the eye is established as a consequence of two events: assignment of the R3 and R4 cell fates, and a rotational movement that carries each ommatidial precursor precisely 90° in opposite directions in the dorsal and ventral halves of the eye (Fig. 1A, larva; reviewed in [1] and [2]). A group of six “core tissue polarity genes,” including *strabismus (stbm)*, also known as *Van Gogh*, ([3], [4]), *frizzled* ([5]–[7]), *disheveled* ([8]–[11]), *prickle* ([12], [13]), *diego* ([14], [15], and *flamingo*, also known as *starry night* ([16]), regulates the establishment of this tissue, or planar cell, polarity by establishing the fates of two photoreceptor cells, R3 and R4. In the “primordial ommatidium,” the cell closer to the equator, the equatorial cell, adopts the R3 cell fate whereas the more polar cell adopts the R4 cell fate (Fig. 1A, larva). Once the fates are determined, ommatidial precursors rotate 90° clockwise in the ventral half and 90° counterclockwise in the dorsal half of the eye, giving rise to the dorso-ventral polarity of the retina. (Reviewed in [1].) (Note that anterior is defined as “to the right” for the preceding and following discussions.)

It is believed that the R3 and R4 cells dictate the direction of ommatidial rotation, although it has not been established that the R3/R4 fates and the direction of rotation are genetically separable. Based on the assumption that cell fates determine the direction of rotation, the correct vs. incorrect direction of rotation is defined here with respect to the R3/R4 fates. An ommatidium that rotates counterclockwise in the dorsal half of the eye is therefore considered to rotate “correctly” (Fig. 1A dorsal, larva).

In eyes mutant for the tissue polarity genes, three phenotypically mutant classes of ommatidia that are relevant to this discussion are evident. The basis of these classes, which are defined by the axis or axes on which they are inverted, lies in the correct vs. incorrect direction of rotation. These classes include: anterior/posterior (A/P) defects, or ommatidia inverted on their A/P axis; anterior-posterior/dorso-ventral (AP/DV) defects, which are ommatidia inverted on both A/P and D/V axes; and dorso-ventral (D/V) defects, in which ommatidia are inverted on their D/V axis. (Symmetric ommatidia, both of the R3/R3- and R4/R4-type, in which an ommatidium adopts two cells of one fate and no cells of the other fate, are also seen in tissue polarity mutant eyes and will be discussed in the results.)

The origin of these defects is a result of both the fates adopted by the R3 and R4 precursors and the direction in which the ommatidia rotate (see also [3]). Ommatidial precursors in which the R3 and R4 fates are reversed, such that the equatorial cell adopts the R4 fate and the polar cell adopts the R3 fate, yet the cluster still rotates appropriately with respect to those fates, gives rise to D/V inversions (Fig. 1B). If this same ommatidium were to instead rotate in the wrong direction with respect to the R3 and R4 fates, the ommatidium's apex would still point in the right direction relative to its dorsal or ventral hemisphere, but the cells that would ordinarily face anteriorly (R1, R2, R3) now face posteriorly, resulting in an A/P inversion (Fig. 1B). In AP/DV inversions, the appropriate fates are adopted, but the ommatidial precursor rotates incorrectly with respect to that fate choice (Fig. 1B). This interpretation of the origin of these subclasses of mutant ommatidia (specifically the A/P and AP/DV defects) supports the hypothesis that cell fate and direction of rotation can be uncoupled, as follows (and as described in [3]). While D/V inversions suggest that fate specification and rotation are linked, the occurrence of A/P and AP/DV inversions suggest that tissue polarity may be a consequence of two distinct events – the asymmetric polarization of each ommatidium due to fate specification and the direction of ommatidial rotation – that are seamlessly coordinated in wild type to give rise to a perfectly patterned retina.

Genes that affect rotation but not cell fate include the genes *nemo* ([17]), *scabrous*([18]) and *EGFR/roulette*([17]–[21]). Ommatidia in flies bearing mutations in these genes consistently rotate in the correct direction; it is only the degree to which they rotate that is disrupted. Therefore, the “rotation genes” do not provide support for the notion that fate and the direction of rotation are genetically separable.

While the observations noted above suggest that fate and rotation are genetically separable events, experimental evidence is lacking. The identification of fate- and direction of rotation-specific genes would support the hypothesis that fate specification and direction of rotation can be genetically uncoupled. If these are distinct genetic events, the disruption of only one pathway by altering the activity of a single gene should result in a modification of specific subclasses of ommatidia in a sensitized genetic background. We carried out a genetic screen to establish if genes with modified activity could affect distinct subclasses of phenotypes (D/V, A/P, or AP/DV) rather than universally affecting all of the subclasses. We conducted a screen of a collection of insertion lines for their ability to dominantly modify a misexpression eye phenotype of *stbm*. We screened a set of GMREP (Glass Multimerized Reporter Enhancer Promoter) lines ([22]; [23]) and identified 86 lines that uniquely modify different subclasses of genes. Notably, of the 11 lines for which we have mapped the insertion site of the P element, none map to the known core polarity genes, so at least a subset of these insertion lines represent pathways that are not core polarity genes, but instead are rotation- or fate-specific. The results reported here affirm that fate and rotation can be genetically separated.

## Materials and Methods

### Dominant Modifier Screen

A collection of approximately 3650 P element insertion lines was screened for modifiers of *stbm*. For the primary screen, male flies from the GMREP collection were crossed to virgin female *sev-stbm* flies at 25°. F_1_ flies were examined under a dissecting microscope for enhancement or suppression of the *sev-stbm/*+ phenotype. Of the lines examined, 164 were identified as enhancers, 34 as suppressors, and an additional eight showed lethal interactions. Males from these 198 lines were crossed again to *sev-stbm* females (as described above) and the F_1_ progeny re-examined at the dissecting microscope level for a modified phenotype. In this “secondary screen,” 51 lines (36 enhancers and 15 suppressors) were eliminated, leaving 147 potential modifiers. Crosses of each of these 147 lines were set up (as described above) and eyes from the F_1_ progeny were sectioned and scored for orientation defects in the “tertiary screen.” For each modifying line, a total of 3 eyes were embedded and an average of 200 ommatidia were scored. A total of 86 modifiers of the *sev-stbm* phenotype were confirmed.

### Statistical Analyses

Fisher's Exact test and the chi-squared test of statistical significance were used to determine the degree of confidence in rejecting the null hypothesis: the (AP+AP/DV) and DV classes will be modified equally by a GMREP line. For the chi-squared test, p-values were calculated with one degree of freedom using the chi dist program in Microsoft Excel. The 2-tail p-value was used in Fisher's Exact test. P-values were identical in both tests. A significant p-value indicates that the DV and (AP+AP/DV) subclasses are not modified to the same degree, but that one class is modified to a significantly greater degree than the other.

## Results and Discussion

### Dominant modifier screen

A dominant modifier screen was conducted to establish whether novel genes, when misexpressed or knocked down, could separate the two events that establish polarity in the *Drosophila* retina: cell fate and the direction of rotation. Since either misexpression or loss-of-function of a gene can equally well address this question, a collection of P element insertion lines, most of which are expected to cause misexpression of genes, was chosen over more traditional approaches for several reasons. First, misexpression of genes in the wrong place, at the wrong time or at greater than endogenous levels can sometimes modify a sensitized phenotype that would not be evident by loss-of-function of the same gene and can therefore sometimes provide insight into a gene's function that cannot be gleaned by other methods. Therefore, the analysis of a collection of misexpression lines may provide a means of establishing whether a gene product can influence a signaling pathway or affect a given process.

We screened for insertion-dependent modification of the *sev-stbm* phenotype. *stbm* is a core tissue polarity gene that encodes a protein with transmembrane domains and a PDZ binding motif ([3]). Misexpression of *stbm* under the control of the *sev* promoter drives high levels of expression of *stbm* in photoreceptors R3, R4, R7 and the cone cells ([24]; [25]). *sev-stbm* flies are viable and have mildly rough eyes when viewed at the dissecting microscope level. In flies bearing one copy of the *sev*-*stbm* transgene, approximately 11% of ommatidia display polarity defects. It is important to note that the *Drosophila* compound eye is so exquisitely patterned that even a single misaligned ommatidium can be detected under the dissecting microscope, indicating this genetic assay is extremely sensitive. While the *sev-stbm* phenotype is only mildly rough, it is sufficiently rough that both suppressors and enhancers can be detected at the dissecting microscope level.

We screened a collection of approximately 3650 lines (kind gift of Bruce Hay, [22]; [23]) in which the P element expression vector, GMREP, was used to drive expression of random genes throughout the genome. In these flies, expression of random genes that lie within 10 kb (upstream or downstream) of the site of insertion of the P element, is driven in all cells behind the morphogenetic furrow. Note that in some cases the P element disrupts gene function, leading to loss-of-function alleles (J. Fetting, unpublished). The lines have been neither systematically mapped nor analyzed. The level of redundancy in the collection is also unknown. The lines in the GMREP collection were tested for their ability to modify the eye phenotype of flies carrying one copy of the *sev-stbm* transgene; 86 modifiers were identified. Notably, only one of these was a suppressor (Rawls *et al.*, in revision).

11% of ommatidia in *sev-stbm* eyes (n = approximately 3,000 ommatidia from 28 eyes) exhibit defects in the D/V, A/P, AP/DV, and the two symmetric classes of ommatidia: R3/R3 and R4/R4 ommatidia (Table 1, Fig. 2B). The majority of defects in *sev-stbm* eyes result from D/V errors (5.5%) and A/P errors (3.7%). Relatively small contributions come from AP/DV (0.7%), R3/R3 (0.7%) and R4/R4 (0.3%) errors. GMREP-modified phenotypes were scored and binned into these subclasses, as illustrated in Table 1. The screen proved to be quite sensitive in that overall enhancement as low as 6.6% (10.9% in *sev-stbm* to 17.5% in GMREP 2594/*sev-stbm*) was detected (Table 1). Furthermore, there may be limits to the number of ommatidia that can be affected, since at most 71.6% of ommatidia were affected (GMREP 136/*sev-stbm*). The observation that no more than 70% of ommatidia are affected in a single eye may be accounted for by redundancy of gene function, or it may simply reflect a randomization that results when the signaling pathway is disabled, in which case some fraction of ommatidia would appear wild-type.

**Table 1:** GMREP modifiers of the *sev-stbm* phenotype

P element	#ommatidia	A/P	D/V	D/V & A/P	R3/R3	R4/R4	Total*
***sev-stbm/+***	**2987**	**3.7%**	**5.5%**	**0.7%**	**0.7%**	**0.3%**	**10.9%**
5	107	21.0%	12.0%	0.9%	6.5%	9.3%	49.7%
43	254	7.1%	28.7%	6.3%	5.5%	6.7%	54.3%
65	173	12.1%	11.0%	5.8%	5.8%	9.8%	44.5%
132	159	7.5%	3.8%	1.9%	30.0%	4.4%	47.6%
136	211	9.0%	19.0%	4.3%	12.3%	13.3%	71.6%
196	225	3.1%	10.2%	8.9%	3.1%	5.3%	30.6%
240	163	11.0%	13.5%	1.2%	9.2%	3.7%	38.6%
288	177	6.8%	11.9%	5.1%	5.6%	5.1%	34.5%
332	228	11.8%	9.2%	3.1%	10.5%	7.9%	42.5%
344	157	10.8%	10.8%	1.3%	8.9%	5.1%	36.9%
352	223	4.5%	21.5%	5.8%	5.8%	9.9%	47.5%
378	215	2.8%	16.3%	13.0%	5.1%	5.6%	42.8%
382	211	5.2%	1.4%	0.5%	8.5%	15.2%	30.8%
415	245	7.8%	15.5%	3.7%	11.8%	10.2%	49.0%
432	138	8.7%	15.2%	5.1%	9.4%	8.7%	47.1%
564	161	13.0%	14.3%	0.0%	0.0%	0.0%	38.5%
**569**	**173**	**0.6%**	**1.2%**	**0.0%**	**0.0%**	**0.0%**	**1.8%**
677	161	4.3%	6.2%	1.9%	21.7%	4.3%	38.4%
**808**	**167**	**22.2%**	**6.0%**	**2.4%**	**1.2%**	**3.0%**	**34.8%**
854	200	4.0%	11.0%	2.5%	7.0%	4.0%	28.5%
910	141	2.1%	15.0%	6.4%	7.1%	11.0%	41.6%
**913**	**172**	**7.0%**	**26.1%**	**0.5%**	**8.1%**	**2.3%**	**44.0%**
929	215	6.0%	14.0%	3.7%	7.0%	3.3%	34.0%
961	110	8.2%	7.3%	4.5%	16.4%	12.7%	49.1%
1048	109	4.6%	10.1%	1.8%	17.4%	11.9%	45.8%
1051	221	9.0%	18.1%	2.7%	6.3%	2.3%	41.1%
1077	152	11.8%	3.9%	0.7%	10.5%	9.9%	36.8%
1185	321	15.0%	19.0%	3.1%	9.0%	12.1%	58.2%
1427	64	6.3%	4.7%	0.0%	18.8%	12.5%	42.3%
1598	110	10.9%	4.5%	0.9%	10.9%	5.5%	32.7%
**1627**	**236**	**7.2%**	**15.3%**	**21.0%**	**6.4%**	**3.8%**	**53.7%**
1648	41	7.3%	7.3%	0.0%	20.0%	0.0%	34.6%
1658	854	9.0%	26.0%	5.4%	8.4%	11.4%	60.2%
1676	199	15.1%	14.6%	1.0%	14.6%	13.1%	58.4%
1771	82	8.5%	2.4%	1.2%	3.7%	6.1%	38.9%
1789	234	6.8%	1.3%	0.0%	3.0%	0.0%	29.5%
1855	215	9.3%	2.8%	0.5%	1.9%	0.5%	23.8%
1881	Missing 1–3 photoreceptors						
1952	170	5.9%	10.0%	0.0%	3.5%	2.9%	29.4%
1967	138	8.7%	8.0%	1.4%	10.1%	0.0%	29.6%
2096	Missing photoreceptors						
2114		10.0%	11.4%	3.3%	14.9%	10.6%	50.2%
**2119**	**222**	**14.0%**	**3.2%**	**0.0%**	**3.6%**	**3.6%**	**30.3%**
**2124**	**98**	**2.0%**	**15.3%**	**1.0%**	**18.4%**	**6.1%**	**43.8%**
2177	211	7.1%	2.8%	1.9%	10.0%	3.8%	29.9%
2192	246	8.1%	13.4%	0.4%	3.3%	3.3%	28.5%
2198	107	1.9%	8.4%	3.7%	1.9%	0.2%	19.8%
2238	130	20.0%	3.1%	0.0%	10.8%	3.8%	42.3%
2254	410	8.9%	24.9%	3.4%	6.8%	3.7%	47.9%
2270	139	5.0%	6.5%	0.0%	20.9%	4.3%	36.7%
2279	101	6.9%	12.9%	3.0%	1.0%	5.0%	47.6%
2282	229	15.7%	10.0%	2.6%	8.3%	4.8%	41.8%
2282	177	8.5%	9.6%	5.6%	8.5%	9.0%	48.0%
2293	256	10.9%	18.0%	2.7%	9.3%	4.7%	46.8%
2296	291	12.7%	12.4%	0.3%	13.1%	6.2%	48.1%
2317	91	4.4%	11.0%	1.1%	18.7%	1.1%	36.3%
2362	Difficult to score; many R3/R3s and R4/R4s						
2364	112	5.4%	11.6%	0.9%	4.5%	2.7%	26.9%
2493	303	9.2%	8.6%	3.6%	11.6%	8.6%	43.3%
2521	172	8.1%	2.9%	0.0%	4.7%	0.0%	22.1%
2523	170	12.9%	7.1%	5.9%	7.6%	10.0%	45.3%
2532	513	7.4%	9.0%	3.1%	3.3%	1.0%	28.5%
2594	144	4.9%	6.3%	0.7%	0.7%	0.7%	17.5%
2601	Missing photoreceptors; many R3/R3s and R4/R4s						
2615	453	8.8%	12.1%	3.0%	12.1%	4.2%	41.9%
2663	361	6.9%	4.7%	2.5%	8.9%	4.7%	28.5%
2681	311	7.7%	2.9%	3.1%	2.3%	6.8%	30.2%
2718	195	10.3%	11.8%	2.0%	12.8%	3.6%	41.5%
2882	205	8.8%	6.8%	4.9%	22.0%	12.2%	59.7%
2885	317	11.7%	11.0%	0.9%	16.1%	11.7%	59.0%
2920	108	8.3%	6.5%	0.0%	8.3%	4.6%	33.3%
2927	168	13.7%	13.1%	2.4%	6.0%	1.2%	40.6%
2971	150	16.7%	9.3%	0.0%	5.3%	10.0%	44.7%
2989	176	8.0%	11.4%	0.0%	12.0%	4.5%	37.6%
3103	Difficult to score; many R3/R3s and R4/R4s						
3107	218	11.0%	14.2%	0.0%	6.0%	4.1%	36.7%
3108	100	8.0%	7.0%	7.0%	13.0%	1.0%	48.0%
3155		12.5%	13.4%	3.6%	10.6%	6.1%	46.2%
3165	335	8.1%	7.5%	4.5%	10.7%	3.0%	37.7%
3185	223	4.9%	6.7%	0.4%	3.6%	2.2%	18.2%
3194	244	4.1%	7.4%	3.3%	9.0%	6.1%	29.9%
3244	337	7.4%	12.8%	5.6%	5.9%	6.5%	40.6%
3295	359	4.7%	12.0%	2.0%	5.6%	6.4%	35.7%
3312	95	21.0%	35.4%	1.0%	5.3%	5.3%	69.0%
3331	100	13.0%	12.0%	2.0%	6.0%	1.0%	35.0%
3356	127	5.4%	6.3%	0.0%	2.4%	11.8%	40.1%
3364	248	9.3%	6.9%	8.9%	3.6%	0.8%	29.9%
3373	235	7.2%	14.5%	1.3%	5.5%	4.7%	35.8%

* Three additional classes were scored (missing R, extra R, failure to rotate) but these data were omitted to simplify the data set. “Total errors” includes defects in these three classes.

Examples in which there was a bias for modification of a single subclass by a GMREP line were identified for all five of the classes of polarity defects scored, including A/P (GMREP 808), D/V (GMREP 913), AP/DV (GMREP 1627), R3/R3 (GMREP 132) and R4/R4 (GMREP 3356) (Table 1, Fig. 2). In certain lines, each of these mutant forms was significantly enhanced relative to the remaining four classes. Neomorphic phenotypes were also observed in some interactions (i.e. GMREP 3356, data not shown), including missing or extra photoreceptors and a failure of ommatidia to rotate.

### Cell fate and direction of rotation can be genetically uncoupled

Both the A/P and AP/DV classes support the theory that cell fate and the direction of rotation can be genetically uncoupled: in both of these classes, ommatidial precursors rotate in the wrong direction with respect to the R3/R4 cell fates. To determine if fate can be separated from direction of rotation, we surveyed the data shown in Table 1 for lines in which the sum of the A/P and AP/DV classes (those classes in which the direction of ommatidial rotation does not follow cell fate) was modified to a significantly greater degree than the D/V class (the class in which the direction of rotation does follow cell fate). Many lines shown in Table 1 exhibit this trend, but only a subset of the strongest enhancers, GMREP 808, GMREP 1627 and GMREP 2119, are discussed in detail. GMREP 808 affects the (A/P+AP/DV) class to a significantly greater extent than it affects the D/V class (wrong R3/R4 fates, right direction of rotation; *p* = 2.08E^−6^). Furthermore, of the A/P and AP/DV classes (wrong direction of rotation), GMREP 808 primarily modifies the A/P class (with respect to the baseline in *sev-stbm*; A/P: wrong R3/R4 fate, wrong direction of rotation) (Table 1; Fig. 2E for representative example). Such an enhancement of the (A/P+AP/DV) class in the relative absence of modification of the D/V class indicates that the direction of rotation is genetically separable from fate choice.

GMREP 2119 acts similarly to GMREP 808: the (A/P+AP/DV) class is significantly more affected than is the D/V class (*p* = 1.6E^−5^) and again, it is primarily the A/P, rather than the AP/DV, class that is modified (Table 1; Fig. 2E for representative example). The gene affected by the P element therefore causes an increase in the number of ommatidia that make the wrong choice regarding the direction of rotation.

As with GMREP 808 and GMREP 2119, the (A/P+AP/DV) class is significantly more affected than is the D/V class in GMREP 1627 (*p* = 3.0E^−4^; Fig. 2D, Table 1). However, in this case, the AP/DV class – not the A/P class – is selectively enhanced. These results again demonstrate that ommatidia do not rely solely on the R3 and R4 cells to control the direction of rotation, again emphasizing the capacity to genetically uncouple fate from rotation.

### Direction of rotation can follow incorrect R3/R4 fate choice

The R3/R4 fates are reversed in the D/V class of ommatidia, yet the direction of ommatidial rotation is consistent with this choice, so while it is unclear if this class necessarily provides evidence that fate and rotation are linked, it certainly does not support the notion that these two events are independently regulated. Significant enhancement of the D/V class of ommatidia relative to the (A/P+AP/DV) class is best illustrated by GMREP 913/*sev-stbm* eyes, in which the D/V class is enhanced relative to the (A/P+AP/DV) classes (*p* = 0.002) (Fig. 2C for representative example; Table 1). GMREP line 2124 provides a second example of a gene that modifies the D/V class to a greater extent than the (A/P+AP/DV) class (p = 0.02)(Table 1). We interpret the disproportionate enhancement of D/V inversions to mean that although there is an early breakdown in fate choice, since ommatidia subsequently rotate correctly with respect to the chosen fates, downstream aspects of the system – those that determine the direction of rotation, for example – can follow the adopted fates.

### Analysis of symmetric defects reveals breakdown of early cell fate decision

While symmetric defects cannot provide further information about the coupling of cell fate and direction of rotation, analysis of the data presented in Table 1 also revealed several notable insights regarding R3/R4 biology. First, the R3 and R4 cells are particularly sensitive to fate changes: the vast majority of GMREP lines tested affect the R3/R4 fate choice, causing a transformation of an R4 to an R3 or an R3 to an R4. In the majority of modified flies, both types of symmetric defects are enhanced to roughly the same degree (Fig. 2F). While either the R3 or R4 class is generally enhanced to a greater degree than the other, there is no clear bias toward one class being enhanced more often than the other. Furthermore, when one of these two classes is dramatically enhanced relative to the other, it is virtually always the R3/R3 class. The specific enhancement of symmetric defects indicates a breakdown in the feedback mechanism that ensures just one cell of each fate is assigned. In summary, these data support the idea that many of the mutations identified in this screen perturb the ability of an ommatidium to make the early decision to produce a single R3 and a single R4 photoreceptor.

The R3 and R4 fates are established by a precisely regulated and finely tuned feedback loop that involves the core tissue polarity genes, the Notch (N) signaling pathway, and a host of additional identified and unidentified participants. Briefly, *fz* activity leads to upregulation of Delta (Dl, the N ligand) transcription in the R3 precursor cell ([26], [27]). Dl then binds to N, stimulating N signaling in the R4 precursor cell. N activity in the R4 precursor inhibits Dl transcription in that cell. The cell with higher N activity adopts the R4 fate ([26]–[28]). The tissue polarity proteins play a critical and dynamic role in regulating Dl transcription in both cells, through what is becoming an increasingly elaborate web of interactions between these proteins.

The hypersensitivity of the R3/R4 fate decision to modification may reflect the complexity of the fate choice (and the feedback loop) compared to the relative simplicity of the decision to rotate clockwise vs. counterclockwise. In other words, more proteins and protein-protein interactions may underlie the fate decision and fewer dictate the decision as to which way the ommatidial precursor rotates – the event that establishes the remaining classes.

Finally, analysis of GMREP 564 indicates that the initial step in assigning one fate can be perturbed yet the feedback loop that ensures each ommatidium has just one R3 and one R4 cell can remain intact. In GMREP 564/*sev-stbm* eyes, the A/P and D/V classes are enhanced, so the initial assignment of the R3 and R4 fates is disrupted, yet neither type of symmetric defect is changed relative to baseline, so the negative feedback loop does not break down.

### Suppression of the *sev-stbm* phenotype

Suppression of the *sev-stbm* phenotype is rare. Of 3650 lines tested, only one was found to suppress the overall *sev-stbm* phenotype (Table 1; Rawls *et al.*, in revision). The paucity of lines that suppress ommatidial polarity could reflect redundancy of gene function and the consequent resiliency to change. The virtual lack of lines that suppress *sev-stbm* could also be a consequence of excessive non-specific gene expression in the GMREP lines, although this possibility is not supported by the results of an independent screen, in which 250 deletion lines also failed to suppress *sev-stbm* ([25]). Alternatively, since the establishment of ommatidial polarity requires the coordination of two events (fate and direction of rotation) to create a single, wild-type outcome, suppression may require simultaneous mutations in more than one relevant pathway. In support of this hypothesis, a similar modifier screen of *sev>nmo* identified roughly equal numbers of enhancers and suppressors (Fiehler and Wolff, in revision). This may reflect the one-dimensionality of the *nmo* phenotype, degree of rotation, as opposed to the multiplicity of the tissue polarity phenotypes.

While the suppression of the *sev-stbm* phenotype is rare, this large data set did reveal a few examples of suppression when this event was scored in individual subclasses. A/P inversions in only six GMREP lines were decreased relative to the *sev-stbm* baseline, and just 14 GMREP lines suppress the D/V class. Interestingly, we found that there is an inverse relationship in the phenotypic modification of the A/P and D/V classes: in all instances in which the D/V class is suppressed, the A/P class is enhanced (n = 13 events), and in all cases in which the A/P class is suppressed, the D/V class is enhanced (n = 5 events). (The single exception in both cases is the suppressor *bdg* (GMREP 569) (Rawls *et al*., in revision). The degree of enhancement and suppression in each of these lines is small, and although the degree of modification generally does not reach statistical significance, the trend is strikingly consistent. The number of incidents is also small, so it is not clear whether the trend is biologically meaningful, yet this inverse co-modification is intriguing.

The suppression of the D/V class in conjunction with an increase in the A/P class likely means that more of the D/V ommatidia lose their ability to rotate in the right direction, thereby adding to the A/P class. Likewise, in the reverse scenario, modification by a GMREP line renders A/P ommatidia better able to choose the correct direction of rotation. This correlation supports the hypothesis that rotation can be genetically uncoupled from fate in that a gain/loss of the ability to choose the right direction of rotation is reflected in an increase in numbers in the D/V and A/P classes, respectively.

### Concluding Remarks

In *stbm* mutants, several aspects of ommatidial polarity are affected, including chirality (a readout of fate) and direction of rotation. Since these two events can be genetically uncoupled, as described here, the tissue polarity genes must directly regulate more than one pathway to set up polarity in the eye. This system is in contrast to the establishment of polarity in the neuromasts on the lateral line of fish, in which *stbm* is required for only one of the two pathways that regulate polarity of these sensory structures. In zebrafish, hair cell orientation is also a consequence of two events: 1) the oriented division of progenitor cells to give rise to a sibling pair of cells, with one of each pair lying on opposite sides of an axis and 2) the orientation of the hair bundles ([29]). Analysis of the *trilobite* mutant in zebrafish (the *stbm* ortholog in fish) showed that the first of these events is not regulated by *stbm*, whereas the second is ([29]).

In *Drosophila*, the tissue polarity genes also orient specialized cuticular structures, the wings and bristles, on various epithelia throughout the fly. Clearly, these genes are required, and are required for distinct and apparently parallel processes, but the underlying molecular and cell biological mechanisms of this regulation are poorly understood. However, what is becoming increasingly clear is that these genes are required in diverse, apparently unrelated ways.
